# Vancomycin and nisin A are effective against biofilms of multi-drug resistant *Staphylococcus aureus* isolates from human milk

**DOI:** 10.1371/journal.pone.0233284

**Published:** 2020-05-29

**Authors:** Angeliki Angelopoulou, Des Field, Mariana Pérez-Ibarreche, Alicja K. Warda, Colin Hill, R. Paul Ross

**Affiliations:** 1 School of Microbiology, University College Cork, Cork, Ireland; 2 APC Microbiome Ireland, University College Cork, Cork, Ireland; Universidade Nova de Lisboa, PORTUGAL

## Abstract

Human milk provides complete nutrition for infants and at the same time promotes the growth of specific bacteria in the infant gastrointestinal tract. Breastfeeding can often be discontinued due to mastitis which is an inflammation of the breast tissue. We isolated 18 *Staphylococcus aureus* strains from milk donated by healthy (n = 6), subclinical (n = 6), and mastitic (n = 6) mothers, two strains of which were VISA (Vancomycin Intermediate *S*. *aureus*). All tested strains (n = 12) were able to form biofilms. We then examined the impact of nisin A and vancomycin alone and in combination on biofilm formation and eradication of selected strains (n = 8). We observed strain-specific responses, with the combinatorial treatment at 1/4X MIC (for both singularly) significantly inhibiting biofilm formation for seven out of eight strains when compared with nisin A or vancomycin alone. None of the selected treatments were able to eradicate pre-formed biofilms. Finally, we selected two strains, namely a VISA (APC3814H) and a strong biofilm former (APC3912CM) and used confocal microscopy to evaluate the effects of the antimicrobial agents at 1X MIC on biofilm inhibition and eradication. All treatments inhibited biofilm formation of APC3814H but were ineffective in eradicating a pre-formed biofilm. Single treatments at 1X MIC against APC3912CM cells did not prevent biofilm formation whereas combination treatment caused increased death of APC3912CM cells. Finally, the combination treatment reduced the thickness of the pre-formed APC3912CM biofilm as compared with the single treatments.

## Introduction

Human milk is composed of macronutrients, vitamins, minerals, and water thus providing optimal nutrition for growth and development of the newborn infant [1]. International health organizations including the World Health Organization (WHO) support exclusive breastfeeding for the first six months of life [2]. The existence of a close relationship between the infant’s gut microbiota and the breast milk microbiota has been reported [3] with the breast milk microbiota seeding the infants’ gastrointestinal (GI) tract [4]. Nonetheless, exclusive breastfeeding may not be an option for mothers for a variety of reasons [5], with mastitis considered the major cause of cessation of breastfeeding. Human mastitis is defined as breast inflammation and affects up to 33% of lactating women [2] and is caused mainly by milk stasis and infection [6]. Mastitis is divided into clinical and subclinical based on the symptoms with clinical mastitis being identified by breast redness, fever, pyrexia, and discomfort while subclinical mastitis has a reduced rate of diagnosis and is characterized by a needling pain and a burning sensation [7]. The standard treatment of mastitis is the administration of antibiotics with penicillinase-resistant penicillins and first-generation cephalosporins being the most suitable antibiotic therapies for the treatment of mastitis [6]. It has been reported that 25% of mothers who stop breastfeeding as a result of mastitis have already received antibiotherapy for 2–4 weeks with little success [8]. In addition, mastitis is often a recurrent or chronic infection due to methicillin resistance and a common ability to form biofilms by *Staphylococcus aureus* and *Staphylococcus epidermidis* which have been identified as the major pathogenic bacteria involved in clinical and subclinical mastitis, respectively [9]. *S*. *aureus* is identified in approximately 20% of the healthy population with the carrier facing a risk of infection [10]. The two aforementioned staphylococci are known for their ability to form biofilms [11] which are structured bacterial consortia entrenched to polymeric substances [12] and can adhere to biotic and abiotic surfaces [13]. Biofilms exhibit intrinsic recalcitrance to antibiotics [12] and are resistant to attacks by phagocytes which are impaired in infiltrating the biofilm matrix [12].

The glycopeptides, and especially vancomycin, are the main treatment modalities for MRSA (Methicillin-resistant *S*. *aureus*) infections [14]. Vancomycin impairs cell wall synthesis by binding to the N-acyl-d-Ala-d-Ala moiety of lipid II which is involved in cell wall biosynthesis and impedes cross-linking of peptidoglycan chains thus leading to cell death [15]. Vancomycin in Ireland is administered as a treatment for mastitis when the women are allergic to penicillin and/or systemically unwell [16, 17].

Due to the increase in antibiotic resistance, bacteriocins have emerged as attractive alternative agents in combination with antibiotics as they can expand the antimicrobial spectra and reduce the required concentration of the antibiotic for effective treatment, thus potentially eradicating side effects [18]. Nisin A is a bacteriocin belonging to the lantibiotics (lanthionine-containing antibiotics) and is composed of 34 amino acids with significant post-translational modifications. Nisin A exhibits antimicrobial activity primarily against Gram positive bacteria while it acts against Gram negative bacteria only when the outer membrane is damaged by other treatments [19]. Nisin A exhibits antimicrobial activity by binding to the pyrophosphate moiety of lipid II [20] impeding the trans-glycosylation step in cell wall biosynthesis and sequesters lipid II from its functional location [21]. Additionally, the nisin-lipid II complex forms pores in the membrane leading to cell death [22].

In this study, we isolated 18 *S*. *aureus* strains from healthy, subclinical, and clinical mastitic human milk samples. We then investigated their diversity, antibiotic resistance, and biofilm formation. Finally, we investigated the effects of nisin A and vancomycin individually and combined on the inhibition of biofilm formation and the eradication of pre-formed biofilms of selected *S*. *aureus* strains.

## Materials and methods

### Bacterial strains

Approximately 10 mL of milk were collected aseptically from 18 lactating women. Six of the participants were healthy (H), six were diagnosed with clinical mastitis (CM) and six with subclinical mastitis (SM). This study was approved by the Cork Clinical Research Ethics Committee. Written informed consent was signed by all the participants in the study.

The sample collection was performed using sterile gloves in a sterile tube with the first few drops (~500 μL) being discarded. Prior to sample collection, nipples and mammary areola were cleaned with sterile alcohol-free aqueous solution moist wipes (Ted Kelleher, First Aid & Hygiene Supplies Ltd, Macroom, Ireland). Samples were stored below 4°C overnight until further processing. Aliquots of 1 mL of milk were subject to cultivation within 24 hours of donation. Tenfold serial dilutions were performed in Maximum Recovery Diluent (MRD; Oxoid, Basingstoke, UK) and 100 μL aliquots were spread-plated onto Baird Parker agar (Oxoid) supplemented with 50 mL egg yolk tellurite emulsion (Oxoid) which selects for staphylococci. All plates were incubated aerobically at 37°C for 48 h. Five colonies were isolated per sample, streaked for purity and grown aerobically in 10 mL of TSB broth (Merck, Germany) at 37°C overnight. The isolated colonies were subjected to colony PCR according to Angelopoulou et al [23] to identify the species of the colonies with 18 of the isolates being identified as *S*. *aureus*. The remaining isolates belonged to *S*. *salivarius* (10/90), *S*. *lugdunensis* (15/90), *S argenteus* (25/90), and *S*. *hominis* (27/90).

### Antibiotic susceptibility testing

The susceptibility of bacterial isolates to antibiotics was tested on Mueller Hinton agar (MH; Oxoid) using the Kirby-Bauer disc diffusion method [24]. Antibiotic sensitivity discs (Oxoid) contained ampicillin (2 μg), cefalexin (30 μg), gentamicin (10 μg), erythromycin (15 μg), clindamycin (2 μg), tetracycline (30 μg), rifampicin (5 μg), benzylpenicillin (1 unit) and erythromycin (15 μg). The selection of the aforementioned antibiotics is based on their use to treat human mastitis [6]. Vancomycin susceptibility is tested only by MIC [25] based on the EUCAST standards as “disk diffusion is unreliable and cannot distinguish between wild type isolates and those with non-vanA-mediated glycopeptide resistance”. According to standard procedures [24], the tested strains were grown to log-phase and spreaded to the dried surface of a MH plate. The antimicrobial discs were placed on each plate and the plates were incubated aerobically for 24 h at 37°C. Zones of growth inhibition around each disc were measured and interpreted by the zone breakpoint standards of EUCAST clinical breakpoint 2019 report [25]. The above experiments were performed in three biological replicates.

### DNA extraction

Genomic DNA was extracted from the 18 *S*. *aureus* strains using the DNeasy Blood and Tissue kit (Qiagen, Hilden, Germany) with slight modifications. For each strain, 2 mL of an overnight culture (Tryptic Soy Broth, TSB, Merck; 1% inoculum) was centrifuged at 14,000 rpm for 10 min and the supernatant was discarded. The cell pellet was resuspended in 180 μL of enzymatic lysis buffer and was incubated for 2 h at 37°C. The remaining steps were performed according to the original protocol. Genomic DNA was quantified using a Qubit dsDNA HS Assay Kit (Invitrogen, ThermoFisher Scientific, Waltham, Massachusetts, USA).

### (GTG)5-PCR

(GTG)_5_-PCR was performed to investigate whether tested isolates were the same or different strain [26]. Amplification was carried out in a 25 μL reaction mixture consisting of 50 ng/μL genomic DNA, 1 μM of the (GTG)_5_ primer (5′-GTG GTG GTG GTG GTG-3′), 12.5 μL of Blue TEMpase Hot Start 2x (VWR, Dublin, Ireland). Amplification was performed in a MiniAmp Plus Thermal Cycler (ThermoFisher Scientific, Waltham, Massachusetts, USA). The PCR conditions applied were: initial denaturation 95°C for 15 min, followed by 35 cycles of 95°C for 30 sec, annealing at 40°C for 40 sec and elongation at 72°C for 30 sec with a final extension step at 72°C for 5 min. The rep-PCR products were electrophoresed in a 1.5% (w/v) agarose gel for 3 h. Banding patterns were visualized using GeneGenius Imaging System (Syngene, Cambridge, UK) and further analysed using GelJ [27]. Dendrogram was generated using the following parameters: similarity method (Dice), linkage (UPGMA) and tolerance of 2%.

### Nisin purification

Nisin A was purified according to previously described protocols [28, 29]. The purified nisin A peptide was subjected to MALDI-ToF Mass Spectrometric analysis to confirm purity before use.

### Minimum inhibitory concentration assays

Minimum inhibitory concentration (MIC) determinations were carried out in three biological replicates in 96-well microtitre plates according to Field and collaborators [30]. The plates were pre-treated with bovine serum albumin (BSA) prior to the addition of the treatments. Briefly, to each well of the microtitre plate, 200 μL of phosphate buffered saline (PBS; Sigma-Aldrich, Germany) containing 1% (w/v) BSA was added and incubated for 30 min. The wells were washed with 200 μL PBS and allowed to dry for 20 min. Nisin A and Vancomycin (Sigma-Aldrich) were resuspended in TSB to a stock concentration of 60 μM and 256 μg mL^-1^, respectively. Nisin A was adjusted to 15 μM (50 μg mL^-1^), while vancomycin to 64 μg mL^-1^ starting concentration and two-fold serial dilutions of each treatment were made in 96 wells plated for a total of eight dilutions. *S*. *aureus* strains were grown overnight in TSB (Merck; 1% inoculum), subcultured into fresh broth and allowed to grow to an OD_600_ of 0.5. The target strain was added after being diluted in 1:100 (10^5^ cfu mL^-1^) in a volume of 200 μL and following incubation for 16h at 37°C, the MIC was read as the lowest peptide and antibiotic concentration causing inhibition of visible growth.

### Biofilm formation

Static microtitre plate assay was performed according to Field et al [31] to investigate the ability of the tested strains to form biofilms. Briefly, TSB supplemented with 1% D-(+)-glucose (ThermoFisher Scientific) (TSBg) was used in this assay which aids in biofilm formation [32]. A 1:100 dilution was performed by adding 2 μL of log phase cells (10^7^ cfu mL^-1^ of each culture) to 198 μL of TSBg in wells of a sterile 96-well microtitre plate (Starstedt, Leicester, UK) giving a starting inoculum of 10^5^ cfu mL^-1^. Additionally, 200 μL of TSBg was used as a negative control. Following incubation at 37°C for 48h to allow for biofilm formation, the plates were removed and gently washed once with 200 μL PBS. Subsequently, 200 μL of methanol was added to fixate the formed biofilms for 15 min. Once fixed, biofilms were stained with 200 μL of 0.05% crystal violet (CV) for 15 min. Excess stain was rinsed off by placing the microplates under running tap water. The microplates were air-dried, and biofilm-bound CV was solubilized in 200 μL of 33% (v/v) glacial acetic acid by shaking at 100 rpm for 30 min. Absorbance was measured at 595 nm using a microtitre plate reader (Molecular Devices Spectramax M3, Sunnyvale, CA, USA). Data obtained in triplicate were calculated and expressed as the mean plus standard deviation. The experiment was performed in three biological replicates. The tested strains were classified as non-adherent, weakly adherent, moderately adherent, and strongly adherent based on the criteria set by Stepanović et al [33] where the cut-off is set as three standard deviations above the mean OD of the negative control.

### Biofilm inhibition

The ability of Nisin A and vancomycin as well as the combination of the two antimicrobial agents to prevent biofilm formation was carried out as described above with the following modifications. Working stocks of nisin A (60 μM) and vancomycin (256 μg mL^-1^) were prepared and used in the study. At the beginning of the assay, nisin A, vancomycin, and combinations of nisin A and vancomycin, were added to the microtiter plate wells at a concentration of 1X, 1/2X and 1/4X, 1/8X MIC in TSBg in conjunction with the microbial cells (10^5^ cfu mL^-1^) and incubated at 37°C for 24 h. *S*. *aureus* isolates alone were inoculated into a fourth set of wells as untreated controls. The plates were removed, gently washed with PBS, and stained with 0.05% with CV as described above. Optical density readings were performed at 595 nm to determine the final biofilm biomass. Data obtained in triplicate were calculated and expressed as the mean plus standard deviation. The experiment was performed in three biological replicates.

### Biofilm eradication

Biofilm formation was performed as described above. Once biofilms were established (48 h) and washed once with PBS, nisin A, vancomycin, and combination of both were added to the microtiter plate wells to a final concentration of 1X, 2X, 4X, and 8X the relevant MIC as previously determined. Following incubation for 24 h at 37°C, the plates were removed, gently washed once with 200 μL PBS, and stained with 0.05% CV as described previously. The optical density readings were taken at 595 nm to determine the final biofilm biomass. Data obtained in triplicate were calculated and expressed as the mean plus standard deviation. The experiment was performed in three biological replicates.

### Confocal Laser Scanning Microscopy (CLSM)

Effect of nisin A, vancomycin, and their combination on inhibition of biofilm formation and eradication of biofilm of *S*. *aureu*s APC3814H and APC3912CM was studied using μ-plate 8-well uncoated microtiter plates (Ibidi, Germany) suited for confocal microscopy applications. For prevention of biofilm formation, *S*. *aureus* isolates (10^5^ cfu mL^-1^) were added together with both single treatment and combinatorial treatments at 1X MIC to the microtiter plate wells. Wells with no antimicrobial agents were used as untreated controls. Plates were incubated at 37°C for 24 h. For eradication testing, biofilms were formed as described above and subsequently wells were rinsed gently once with 200 μL PBS to remove sessile cells. Nisin A, vancomycin, and their combination were added to the microtiter plates at 1X MIC and the plates were incubated at 37°C for 24 h. A well with pre-formed biofilm was left untreated as a negative control.

Following 24 h antimicrobial treatment, the wells were rinsed gently with 200 μL PBS and stained using a Live/Dead BacLight viability kit (Invitrogen). The microtiter plates were incubated at room temperature for 15 min in the dark. After incubation, residual stain was removed. The images were observed using a Zeiss LSM 5 confocal microscope with EC Plan-Neofluar 20x/0.5 M27 lens and images were acquired using the Zen 3.0 software. Sample was excited using laser light at 488 nm with emission light filtered with a bandpass filter at 475–525 nm for Syto 9 and a longpass filter at 650 nm for propidium iodide (PI). Images were acquired using two distinct confocal channels with pinhole adjusted to 1 at 512 x 512 pixels.

### Statistical analysis

All statistical analyses were performed using SPSS, release 26.0. When data were normally distributed, one-way ANOVA was performed in combination with bonferroni post hoc analysis to evaluate differences between the treatments. In contrast, where data were not normally distributed, Kruskal-Wallis one-way ANOVA was performed in conjunction with Mann-Whitney U post hoc analysis. For all tests, differences were considered significant at p ≤ 0.05.

## Results

### Antibiotic susceptibility testing

Antibiotic susceptibilities of the 18 *S*. *aureus* strains isolated from healthy, subclinical, and clinical mastitic human milk samples from lactating women were evaluated using the Kirby Bauer disc diffusion method for a range of antibiotics representing penicillins, cephalosporins, lincosamides, aminoglycosides, macrolides, and tetracyclines. Of the 18 isolates tested, only two isolates were susceptible to the tested antibiotics which were chosen on the basis of their importance for treating mastitis and their ability to provide diversity for representation of different antimicrobial agent classes (Table 1). More specifically, APC3822H was susceptible to rifampicin and benzylpenicillin while APC3774SM was susceptible to rifampicin, ampicillin, and cefalexin (Table 1).

**Table 1:** Antibiotic susceptibility testing of *S*. *aureus* strains isolated from healthy (H), subclinical (SM) and clinical mastitic (CM) lactating mothers.

	Rifampicin	Benzylpenicillin	Ampicillin	Cefalexin	Clindamycin	Gentamycin	Erythromycin	Tetracycline
**APC3822H**	S	S	R		R			
**APC3819H**	R	R						
**APC3821H**								
**APC3820H**								
**APC3812H**								
**APC3814H**								
**APC3774SM**	S		S					
**APC3809SM**	R		R					
**APC3887SM**								
**APC3813SM**								
**APC3885SM**								
**APC3886SM**								
**APC3815CM**								
**APC3913CM**								
**APC3914CM**								
**APC3911CM**								
**APC3910CM**								
**APC3912CM**								

(R): Resistant; (S): Susceptible

### (GTG)5-PCR

**Fig 1:**
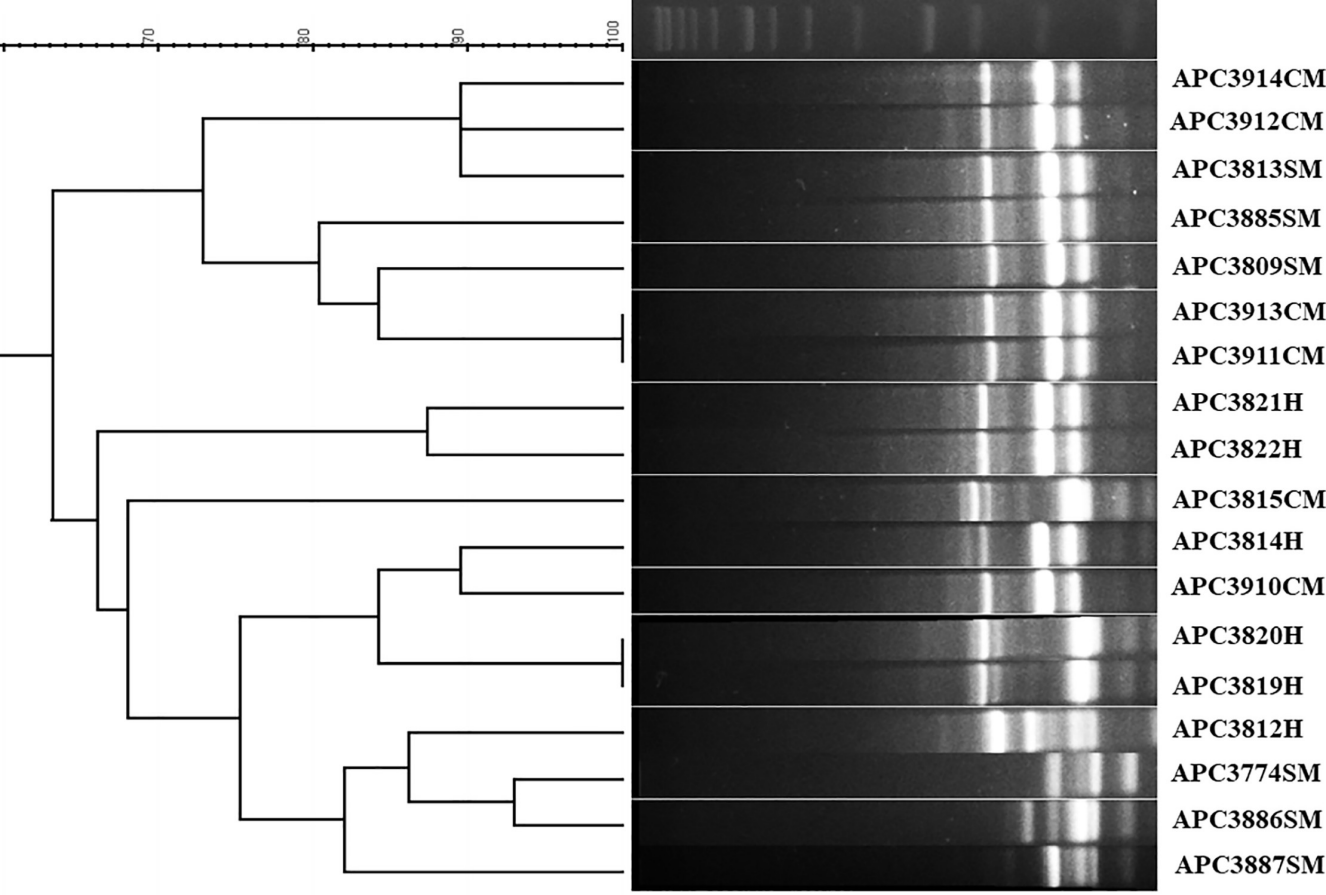
Dendrogram based on the cluster analysis of the (GTG)_5_-PCR fingerprint profiles for 18 *S*. *aureus* strains isolated from healthy (H), subclinical (SM), and clinical (CM) mastitic lactating mothers. The DNA ladder is also presented.

All analysed *S*. *aureus* isolates were typeable using the (GTG)_5_ primer and generated PCR products ranging from 250 to 2500 bp (Fig 1). The dendrogram indicates that the majority of the tested isolates are potentially distinct strains (from each other) with the exception of *S*. *aureus* APC3819H and APC3820H which exhibit identical band patterns and *S*. *aureus* APC3913CM and APC3911CM which also have identical band patterns (Fig 1). Cluster analysis of the (GTG)_5_-PCR fingerprints showed that the tested *S*. *aureus* isolates form two clusters, one containing the majority of the isolates from clinical mastitis and two isolates from subjects with subclinical mastitis and the other one comprises the isolates from healthy subjects, two from clinical mastitis, and four from subclinical mastitis.

### MIC-based investigations of nisin A and vancomycin

Having established that only two of the 18 *S*. *aureus* strains were susceptible to three of the antibiotics, we investigated the MICs of vancomycin and nisin A on four randomly chosen strains, using a random number generator, isolated from individuals in each category: healthy (H), subclinical mastitis (SM), and clinical mastitis (CM). Nisin A is the prototypical bacteriocin with known antimicrobial activity against Gram-positive bacteria while vancomycin is prescribed against MRSA strains. The majority of the tested strains were susceptible to vancomycin with a MIC ≤ 2 μg mL^-1^ (Table 2). *S*. *aureus* ACP3814H and APC3887SM demonstrated intermediate resistance to vancomycin with the MIC being recorded at 4 μg mL^-1^ (Table 2). Regarding nisin A, the *S*. *aureus* isolates exhibited a MIC range of 1.875 μg mL^-1^ to 15 μg mL^-1^ (Table 2).

**Table 2:** MICs for Nisin A and vancomycin for *S*. *aureus* strains isolated from healthy (H), subclinical (SM), and clinical mastitic (CM) lactating mothers.

	APC3814H	APC3820H	APC3821H	APC3812H	APC3774SM	APC3885SM	APC3887SM	APC3813SM	APC3910CM	APC3912CM	APC3913CM	APC3914CM
**Nisin A**	7.5 ug mL^-1^	7.5 ug mL^-1^	3.75 ug mL^-1^	7.5 ug mL^-1^	7.5 ug mL^-1^	3.75 ug mL^-1^	15 ug mL^-1^	7.5 ug mL^-1^	3.75 ug mL^-1^	1.875 ug mL^-1^	7.5 ug mL^-1^	1.875 ug mL^-1^
**Vancomycin**	4 ug mL^-1^	2 ug mL^-1^	2 ug mL^-1^	2 ug mL^-1^	2 ug mL^-1^	1 ug mL^-1^	4 ug mL^-1^	2 ug mL^-1^	1 ug mL^-1^	1 ug mL^-1^	1 ug mL^-1^	2 ug mL^-1^

As the *S*. *aureus* isolates under investigation were susceptible to vancomycin, albeit at varying concentrations, it was selected together with nisin A to determine their effects alone and in combination against preformed biofilms and their potential to impede biofilm formation.

### Biofilm formation

**Fig 2:**
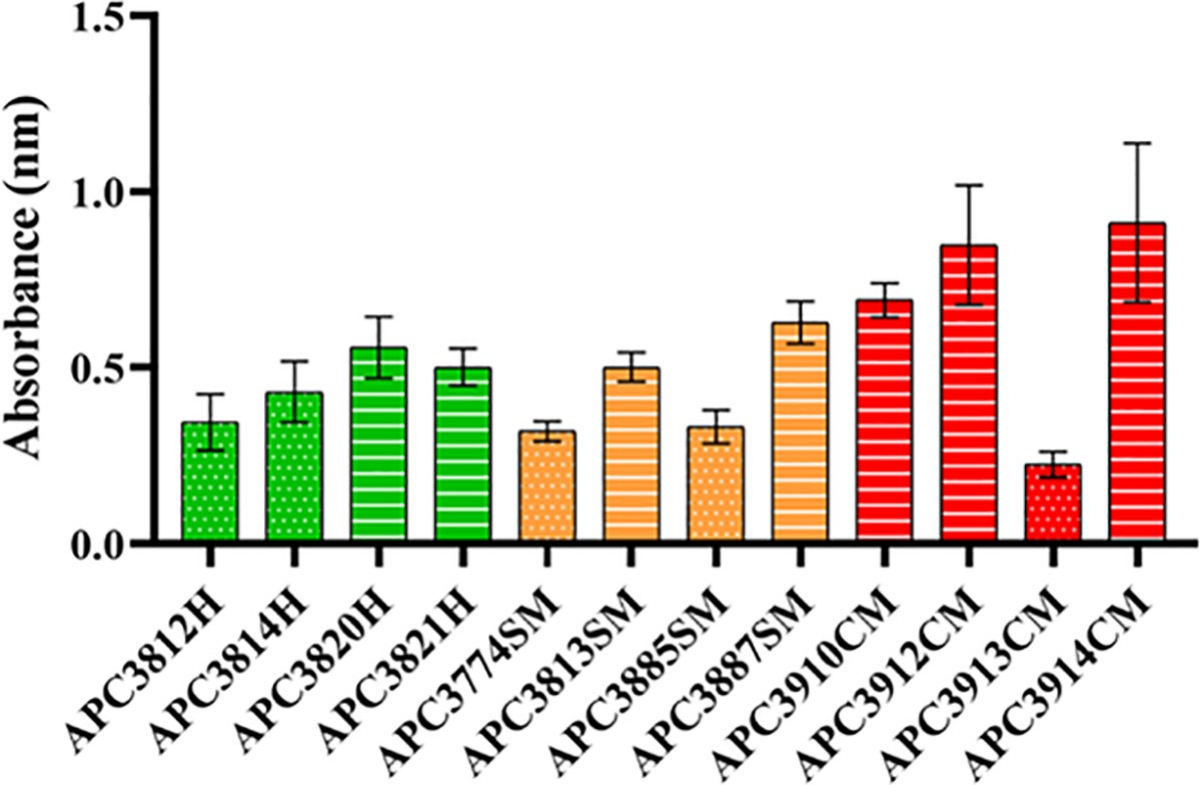
**Biofilm forming ability of *S*. *aureus* strains isolated from healthy (H; green), subclinical (SM; orange), and clinical (CM; red) mastitic lactating mothers.** The amount of biofilm was quantified by measuring the OD_595_ of crystal violet dissolved in acetic acid. The mean ± standard deviation of triplicate biological determinations is presented. Dots depict moderate biofilm formers while lines represent strong biofilm formers.

Biofilm formation is recognized as one of the important virulence factors in staphylococci. The microtiter plate is one of the most commonly utilized techniques to quantify their ability to form biofilms. Prior to initiating studies with nisin A and vancomycin, we evaluated the ability of the 12 selected strains to form biofilms with the CV method. Most strains were able to form strong biofilms, while *S*. *aureus* APC3812H, APC3814H, APC3774SM, APC3885SM, and APC3913CM were moderate biofilm formers (Fig 2).

### Evaluation of nisin A, vancomycin and their combinations against biofilm inhibition

In order to determine the ability of nisin A, vancomycin, and combinations to inhibit biofilm formation we investigated biofilm formation in the presence of inhibitory and sub-inhibitory concentrations of each antimicrobial alone and in combination. Eight of 18 strains were selected, including the VISA strain *S*. *aureus* APC3814H, based on the MIC of nisin as in our opinion it is a good representation to show any strain differences that might be encountered. Five of the eight strains were isolated from subclinical mastitic women and three strains were isolated from healthy women.

**Fig 3:**
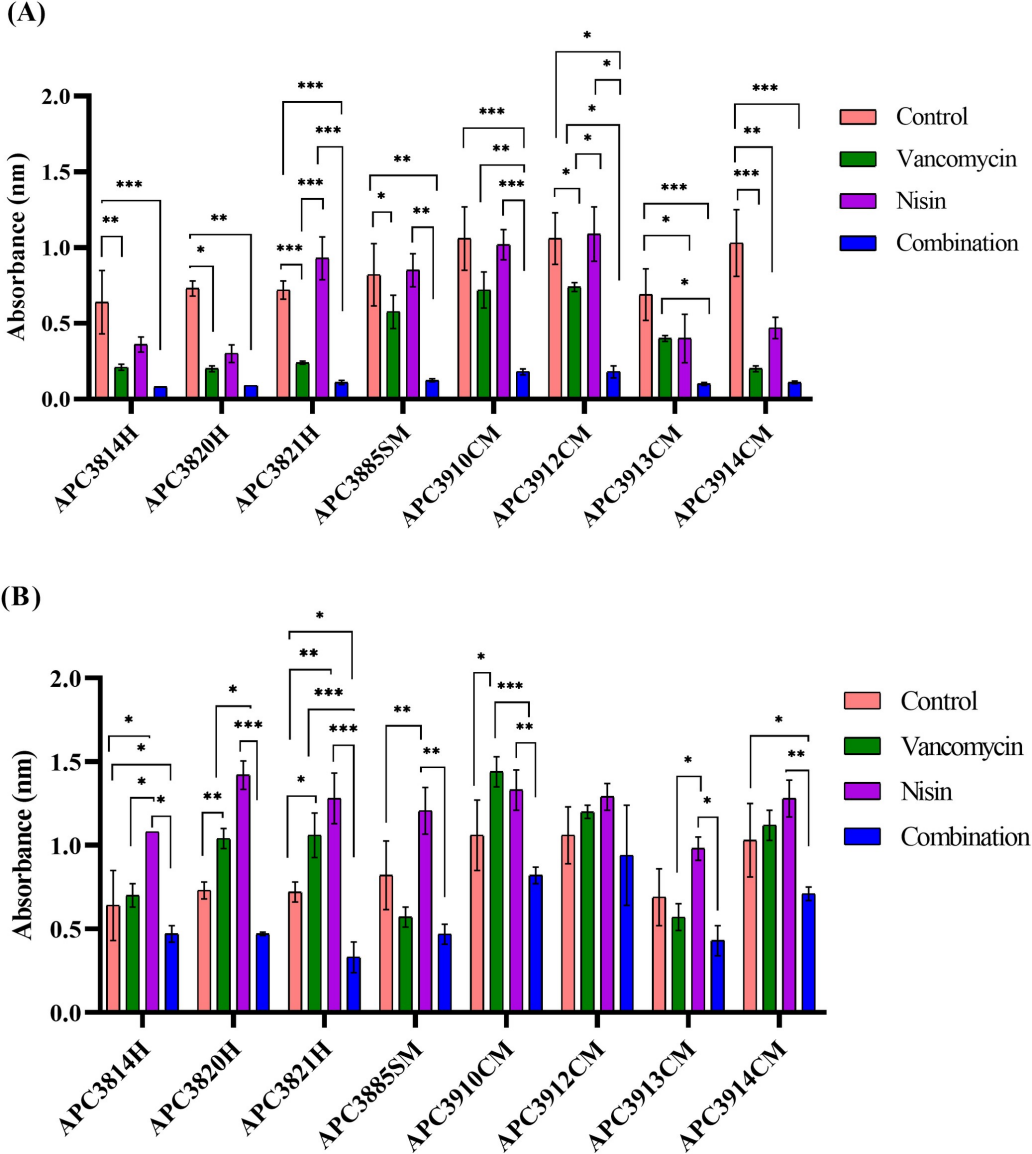
Inhibition of biofilm formation with nisin A, vancomycin and their combination. Results of 24 h treatment of *S*. *aureus* strains, isolated from healthy (H), subclinical (SM), and clinical mastitic (SM) lactating mothers, with 1X (A) and 1/4X (B) MIC of nisin A, vancomycin, and their combination. The amount of biofilm was quantified by measuring the OD_595_ of crystal violet dissolved in acetic acid. The graphs represent the mean of the OD_595_ values from three biological replicates with standard deviations. Asterisks indicate statistically significant differences between treatments and between treatments and control for each strain (* = p ≤ 0.05; ** = p ≤ 0.01, and *** = p ≤ 0.001).

We observed that the combination treatment at 1X MIC was able to significantly inhibit biofilm formation (p ≤ 0.001 for APC3814H, APC3821H, APC3910CM, APC3913CM, and APC3914CM; p ≤ 0.01 for APC3820H and APC3885SM; p ≤ 0.05 for APC3912CM) compared with the control (no treatment; Fig 3A). When we investigated the effect of vancomycin at 1X MIC, we observed a similar outcome in that vancomycin significantly impeded biofilm formation for most of the strains (p ≤ 0.001 for APC3821H and APC3914CM; p ≤ 0.01 for APC3814H; p ≤ 0.05 for APC3820H, APC3885SM, APC3912CM) with the exception of *S*. *aureus* APC3910CM and APC3913CM where vancomycin had no significant effect on inhibition of biofilm formation (Fig 3A). Nonetheless, nisin A had no effect on biofilm formation for *S*. *aureus* APC3821H, APC3885SM, APC3910CM, and APC3912CM.

When we examined the effects of the treatments on biofilm formation at 1/4X MIC, we observed that the combinatorial treatment significantly inhibited biofilm formation for *S*. *aureus* APC3814H, APC3821H, and APC3914CM (p ≤ 0.05; Fig 3B). In contrast, treatment of the forming biofilms with either 1/4X MIC nisin A or vancomycin resulted in significantly increased biofilm formation for most of the tested strains with the exception of APC3912CM where the treatments had no significant effects (Fig 3B). A similar pattern was observed at 1/8X MIC where all tested antimicrobials had no significant effect on biofilm inhibition (S1 Fig) while single treatments stimulated biofilm formation. At 1/2X MIC, the combination treatment significantly suppressed biofilm formation for *S*. *aureus* APC3821H (p ≤ 0.01), APC3885SM (p ≤ 0.05), and APC3914CM (p ≤ 0.001) and outperformed either nisin A or vancomycin alone (S1 Fig).

### Evaluation of nisin A, vancomycin and their combinations for biofilm eradication

**Fig 4:**
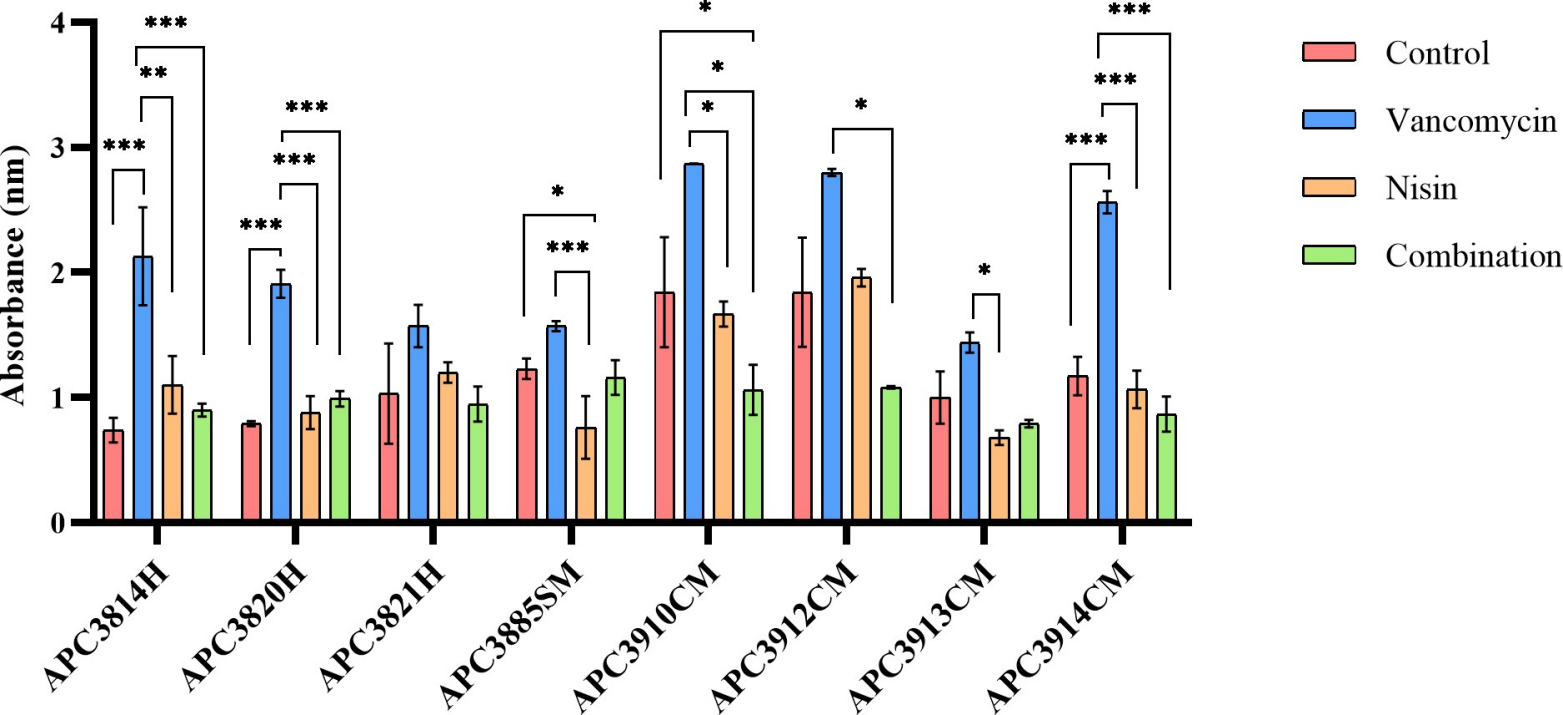
Eradication of pre-formed *S*. *aureus* biofilms with nisin A, vancomycin and their combination. Results of 24 h treatment of *S*. *aureus* strains, isolated from healthy (H), subclinical (SM), and clinical mastitic (SM) lactating mothers, with 4X MIC of nisin A, vancomycin, and their combination as evaluated by crystal violet straining. The amount of biofilm was evaluated by measuring the OD_595_ of crystal violet dissolved in acetic acid. The graphs represent the mean values from three biological replicates with standard deviations. Asterisks indicate statistically significant differences between treatments and between treatments and control for each strain (* = p ≤ 0.05; ** = p ≤ 0.01, and *** = p ≤ 0.001).

Pre-formed biofilms of the selected *S*. *aureus* strains on a 96-well plate were incubated with nisin A, vancomycin, and their combinations at concentrations of 1X, 2X, 4X, and 8X for 24 h (Fig 4 (4X MIC) and S2 Fig). In the case of *S*. *aureus* APC3814H, APC3820H, and APC3914CM, treatment with vancomycin at 4X MIC led to significant increases of biofilm mass compared with the untreated control (p ≤ 0.001; Fig 4). In contrast, nisin A significantly decreased biofilm mass for *S*. *aureus* APC3885SM (p ≤ 0.05). The combination treatment had no significant effect on reducing the biofilm mass for the tested strains with the exception of APC3910CM where combination treatment at 4X significantly reduced the biofilm mass (Fig 4). It should be highlighted that even at high concentrations (8X MIC) of the selected treatments no biofilm eradication was observed. Furthermore, the treatments at this concentration and especially vancomycin were unable to significantly reduce the biofilm mass of the tested strains (S2 Fig).

### Analysis of the impact of nisin A, vancomycin and their combinations on biofilm inhibition and eradication using CLSM

To further assess the impact of nisin A, vancomycin, and their combination at 1X MIC on inhibition of biofilm formation and eradication of pre-formed biofilms, we visualized the biofilms using CLSM conjointly with the BacLight Live/Dead staining kit which enables differentiation between live and dead cells. We selected one VISA strain (APC3814H) and one strong biofilm former isolated from mastitic human milk (APC3912CM).

**Fig 5:**
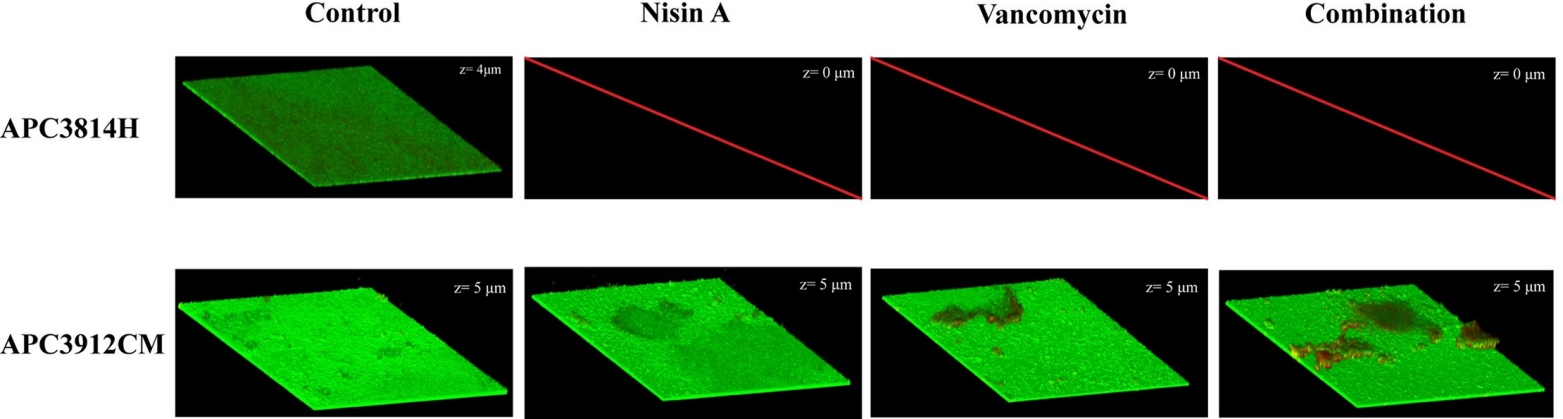
Live/dead 3D CLSM images of inhibition of biofilm formation for *S*. *aureus* APC3814H and *S*. *aureus* APC3912CM. The biofilms were assayed for inhibition after 24 h of treatment. The images were acquired from the centre of the well. z: thickness of the biofilm (μm).

Following 24 h in the presence of antimicrobials, all treatments were able to inhibit biofilm formation of *S*. *aureus* APC3814H (Fig 5). However, we did not observe the same effect for APC3912CM. The biofilm thickness of the untreated control of APC3912CM did not differ significantly from those of the treatment groups (z = 5 μm; Fig 5). Following vancomycin treatment, we observed increased numbers of dead cells in the centre of the well of APC3912CM (Fig 5) that could be associated with a better capacity of vancomycin to diffuse through this area of biofilm. When the combination treatment was applied, we recorded a significant kill effect compared with vancomycin alone suggesting a synergistic effect of nisin and vancomycin (Fig 5). The same phenomena were observed for the edge of the well (S3 Fig).

**Fig 6:**
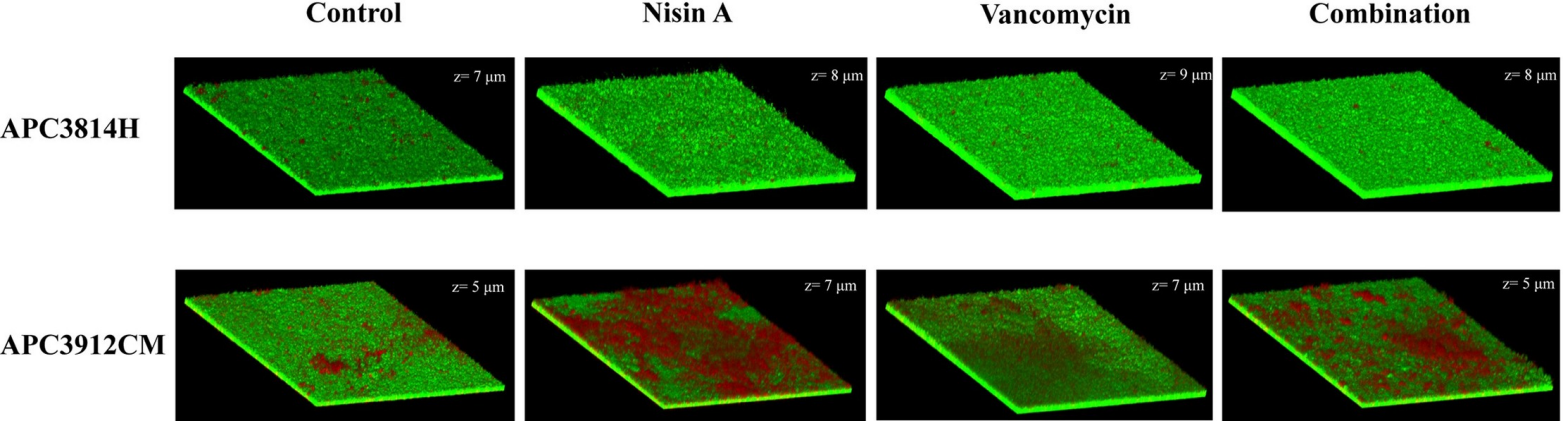
Live/dead 3D CLSM images of biofilm eradication for *S*. *aureus* APC3814H and *S*. *aureus* APC3912CM. The biofilms were assayed for inhibition after 24 h of treatment. The images were acquired from the centre of the biofilm. z: thickness of the biofilm (μm).

Subsequently, we investigated whether the selected treatments could eradicate pre-formed biofilms over 24 h. The untreated biofilm of *S*. *aureus* APC3814H had dead cells in the centre of the well and a thickness (z) of 7 μm (Fig 6). When the treatments were applied to the pre-formed biofilms, fewer dead cells were observed in the centre of the wells. Treatment of the biofilm with nisin A increased the biofilm thickness by 1 μm while vancomycin treatment led to a thickness increase by 2 μm (z = 9 μm) confirming the results of the microtiter plate assay (Fig 6). We also examined the top left edges of the wells since we noticed significant death at this site in the untreated control compared to the control centre, with the thickness of the former recorded at 4 μm (S4A Fig). After applying the treatments, a slight increase in the biofilm thickness at the top left edges was noticed (z = 6 μm). Following vancomycin and combinatorial treatment, significant death was recorded compared to treatment with nisin A (S4B–S4D Fig). *S*. *aureus* APC3912CM control also exhibited some death in the centre of the well with the thickness of the biofilm recorded at 5 μm (Fig 6). However, when the biofilm was treated with nisin A, a significant number of dead cells were detected in the middle of the biofilm (z = 7 μm; Fig 6). Vancomycin treatment led to slightly increased levels of death and to increased biofilm thickness (z = 7 μm; Fig 6). When treatments were combined, a two μm decrease of the biofilm thickness (to 5 μm) was recorded with significant death in the middle of the well (Fig 6). During inspection of the edge of the well, we recorded the same thickness as the middle of the well and similar numbers of dead cells as the centre (S5A Fig). Treatments did not influence the thickness of the biofilm at the edge of the well, and after vancomycin treatment we did not observe an apparent difference regarding the number of dead cells in the centre of the well compared with the edge (S5B–S5D Fig).

## Discussion

Staphylococci and more specifically *S*. *aureus* and *S*. *epidermidis* have been identified as major pathogens in human mastitis [8; 34]. *S*. *aureus* forms biofilms, complex structured bacterial consortia that increase the antimicrobial resistance of the microorganism and facilitate the infectious process [35]. Indeed, antibiotic resistance is widespread in the genus, further complicating treatment options. Lately, due to the extensive increase of antibiotic resistance, major efforts have been put in to identify and develop antimicrobial agents effective against these multi-resistant bacteria. Lantibiotics are in the spotlight because of their high potency *in vitro* and their ability to swiftly impair target cells [36, 37]. Furthermore, lantibiotics are amenable to bioengineering [38] which could introduce a novel arsenal of antimicrobial agents. Nisin A is a model lantibiotic with ability to penetrate the biofilm matrix [39] and proven efficiency against biofilms of *S*. *aureus* (MRSA included) [40]. More specifically, bioengineering has proved effective in creating nisin derivatives with enhanced antimicrobial activity against both non-pathogenic [41] and pathogenic [30; 42, 43] targets. More recently, Ellis et al [44] have demonstrated that the combination of nisin Z with methicillin was more effective against MRSA when compared to single treatments, revealing partial synergy and significantly reduced staphylococcal counts on porcine skin.

Fernández and collaborators [45] are the only group to date that have investigated nisin A as an alternative treatment for human staphylococcal mastitis. The study comprised two groups of four women each where the first group received a solution of nisin A applied to the nipple and mammary areola whereas the second group received a solution with no nisin. By the end of the study (day 14), the average counts in the nisin group were significantly reduced (~2 log cfu mL^-1^) compared to the control group with the clinical symptoms in the nisin group being eliminated.

Here, we endeavoured to study the effects of nisin A in combination with vancomycin on *S*. *aureus* strains isolated from human milk obtained from healthy, subclinical, and mastitic women. Vancomycin is the cornerstone therapy against antibiotic-resistant *S*. *aureus* and is clinically used to treat MRSA infections. Consequently, the failure of vancomycin to control *S*. *aureus* infections may lead to a major public health issue.

Only two of 18 tested strains were susceptible to cephalexin, a first-line therapy for lactational mastitis that is administered in cases of penicillin intolerance [45]. All tested strains were resistant to erythromycin and clindamycin which is of interest given that both drugs are prescribed to treat lactational mastitis [46]. More specifically, clindamycin is prescribed when the patient is allergic to penicillin and in cases of recurrent mastitis [47]. Previous studies have also reported the isolation of antibiotic-resistant *Staphylococcus* strains from human milk of healthy [48–50] and mastitic donors [36]. To our knowledge, this is the first time in literature, that such extent of drug resistance is reported in human milk. These findings demonstrate the extent of antibiotic resistance in human milk strains and reinforce the need for new therapeutic approaches, especially since human milk seeds the GI tract of infants [4; 51], thus further exacerbating the problem of antibiotic-resistance. Two of the tested strains were vancomycin-intermediate resistant potentially supporting the MIC drift phenomenon generated by the extensive use of vancomycin that has elevated its MIC [52]. The MIC range for nisin A recorded in our study (1.875–15 ug mL^-1^) was in close agreement with the MIC range for nisin A reported by Dosler and Gerceker [40].

All tested strains were able to form biofilms with the majority being strong biofilm formers and only APC3812H, APC3814H, APC3774SM, APC3885SM, and APC3913CM being recorded as moderate biofilm formers (Fig 2). This could prove a major problem in successfully treating mastitis. During the biofilm inhibition assays, we observed that sub-inhibitory concentrations of vancomycin (1/8X MIC) enhanced biofilm formation in the tested strains with the exception of APC3885SM, APC3912CM, APC3913CM, and APC3914CM confirming the findings of previous studies for some clinical isolates of *S*. *epidermidis* [53], for VISA strains [54] and MRSA [55] where vancomycin also stimulated biofilm formation. Combinatorial treatments at 1/4X MIC (Fig 3B) significantly outperformed nisin A and/or vancomycin alone for the majority of the tested strains with the exception of APC3912CM, indicating that combinations of antimicrobial agents have greater potential than single treatments to prevent biofilm formation, and at the same time suggesting a potentially synergistic effect of nisin A with vancomycin. While nisin A at 1X MIC inhibited biofilm formation for the majority of the tested strains (Fig 3B), at sub-inhibitory concentrations some of the strains continued to form biofilms; e.g. APC3820H at 1/4X MIC. This finding is in agreement with work published by Sudagidan and Yemenicioğlu [56] who demonstrated that the growth of 25 *S*. *aureus* strains isolated from milk and cheese was inhibited by nisin A at 25 μg mL^-1^. However, when they investigated the effect of lower nisin A concentration on biofilm inhibition, they recorded a strain-dependent resistance. Resistance of *S*. *aureus* against nisin A has been ascribed to its reduced hydrophobicity and elevated net positive charge following contact with this lantibiotic [57]. In addition, use of the combination treatment at 1X MIC significantly inhibited biofilm formation for the tested strains compared to single treatments which is in accordance with the findings of Dosler and Gerceker [40] who observed synergy for 2 *S*. *aureus* isolates between nisin and vancomycin at 5X MIC. To our knowledge, there are no studies examining subinhibitory concentrations of nisin A and vancomycin and their effect on biofilm inhibition suggesting that future work could be performed in that field.

When the impact of the selected antimicrobial agents against pre-formed biofilms was examined, the vancomycin concentration was increased up to 8X MIC as the usage of higher vancomycin concentrations is not clinically applicable. More specifically, the reference range for vancomycin trough levels is 10–20 μg mL^-1^ whereas the reference range for vancomycin peak levels is 25–50 μg mL^-1^ [58]. At all tested concentrations of vancomycin, the strains continued to form biofilms suggesting that a higher concentration should be used to eradicate the biofilm and at the same time indicating that future studies should establish MICs for biofilms and not planktonic bacteria. One hypothesis to interpret the reduced activity of antimicrobials on biofilms is the predominance of persister cells which are bacterial clones formed stochastically in microbial populations. Persister cells express a distinct yet reversible phenotype that permits them to transiently evade the effects of antibiotics and restore biofilm populations after antibiotic removal [59, 60]. A second explanation is that vancomycin demonstrates limited penetration in *S*. *aureus* and *S*. *epidermidis* biofilms [61, 62]. Combinations of nisin A and vancomycin significantly reduced the mass of pre-formed biofilms for most of the tested strains with the exception of APC3821H compared with single treatments of vancomycin or nisin A alone. However, applied treatments were ineffective at eradicating the established biofilms which is in agreement with the findings of Nishimura et al. [63].

CV staining reveals information about biofilm mass but provides no information on viability of cells. When we investigated the effects of the selected treatments on inhibition of biofilm formation using CLSM, we observed a strain-specific response as biofilm formation of *S*. *aureus* APC3814H was inhibited by all treatments while the same did not apply to APC3912CM. This illustrates the diversity of the tested strains. In terms of biofilm eradication, for *S*. *aureus* APC3814H none of the applied treatments at 1X MIC were able to reduce the thickness of the biofilm which is in accordance with the findings by Okuda et al. [39]. This was not the case for *S*. *aureus* APC3912CM where we observed that the combinatorial treatment reduced the biofilm mass compared to single treatment and caused significant death compared with the untreated control. In addition, for *S*. *aureus* APC3912CM an enhanced penetration and killing effect was noticed when nisin A was applied on the pre-formed biofilm. The ability of nisin A to penetrate the biofilm matrix of several strains of *S*. *aureus* (including MRSA) has been already shown in literature [39] and is in agreement with our finding.

In conclusion, we assessed the effects of nisin A and vancomycin alone and in combination on inhibiting biofilm formation and eradicating pre-formed biofilms with a view to bypassing antimicrobial resistance and increasing antimicrobial efficiency against 18 *S*. *aureus* strains isolated from healthy, subclinical, and clinical mastitic lactating women. A strain-specific behaviour was recorded with combinatorial treatments outperforming either nisin A or vancomycin alone in biofilm inhibition for five out of eight tested strains and biofilm eradication for half of the evaluated strains, implicating that combinatorial treatments could be the next step in eliminating and hampering the formation of problematic biofilms and related infections.

## Supporting information

S1 Fig Inhibition of biofilm formation with nisin A, vancomycin and their combination. Results of treatment of *S*. *aureus* strains, isolated from healthy (H), subclinical (SM) and clinical mastitic (SM) lactating mothers, with 1, 1/2, ¼, and 1/8X MIC of nisin A, vancomycin and their combination for 24 h prior to biofilm formation. The amount of biofilm was quantified by measuring the OD_595_ of crystal violet dissolved in acetic acid. The graphs represent mean values from three biological replicates with the standard deviations. Asterisks indicate statistically significant differences between treatments and between treatments and control for each strain (* = p ≤ 0.05; ** = p ≤ 0.01, and *** = p ≤ 0.001).
(TIF)

S2 Fig Treatment of biofilms with nisin A, vancomycin, and their combination. *S*. *aureus* strains, isolated from healthy (H), subclinical (SM), and clinical mastitic (SM) lactating mothers, with 1, 2, 4 and 8X MIC of nisin A, vancomycin and their combination for 24h as evaluated by crystal violet straining. The amount of biofilm was evaluated by measuring the OD_595_ of crystal violet dissolved in acetic acid. The graphs represent mean values from three biological replicates with standard deviations. Asterisks indicate statistically significant differences between treatments and between treatments and control for each strain (* = p ≤ 0.05; ** = p ≤ 0.01, and *** = p ≤ 0.001).
(TIF)

S3 Fig Live/dead 3D CLSM images of *S*. *aureus* APC3912CM. The biofilm was assayed for inhibition after 24 h of treatment; (A) untreated control, (B) 1X nisin A, (C) 1X vancomycin and (D) 1X nisin A + 1X vancomycin. The images were acquired from the edge of the well.
(TIF)

S4 Fig Live/dead 3D CLSM images of the biofilm of *S*. *aureus* APC3814H. The biofilm was assayed for eradication after 24 h of treatment; (A) untreated control, (B) 1X nisin A, (C) 1X vancomycin and (D) 1X nisin A + 1X vancomycin. The images were acquired from the edge of the well.
(TIF)

S5 Fig Live/dead 3D CLSM images of *S*. *aureus* APC3912CM. The biofilm was assayed for eradication after 24 h of treatment; (A) untreated control, (B) 1X nisin A, (C) 1X vancomycin and (D) 1X nisin A + 1X vancomycin. The images were acquired from the edge of the well.
(TIF)

S1 Raw images (TIF)
