# Supplementary figures and images for: Vancomycin and nisin A are effective against biofilms of multi-drug resistant Staphylococcus aureus isolates from human milk
Source: PLoS One. 2020 May 29;15(5):e0233284. doi: 10.1371/journal.pone.0233284 (PMC7259672; doi:10.1371/journal.pone.0233284)

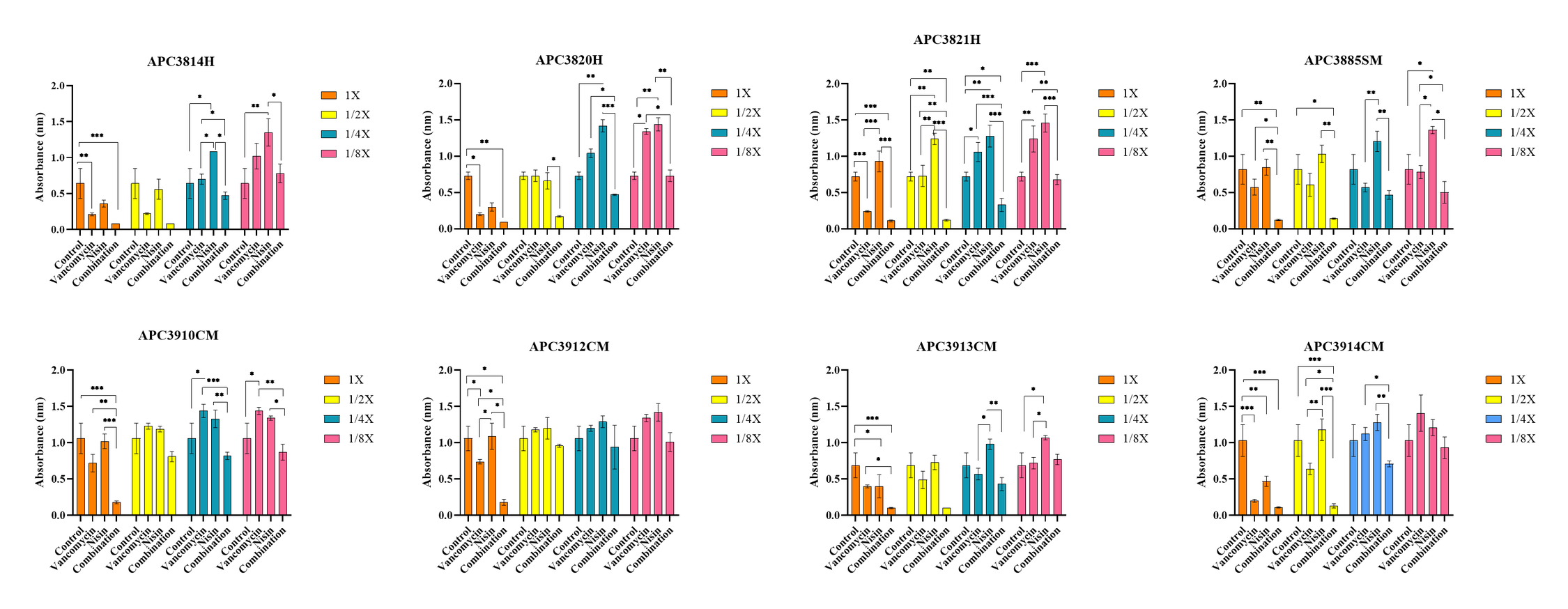

Supplement: S1 Fig — Results of treatment of S. aureus strains, isolated from healthy (H), subclinical (SM) and clinical mastitic (SM) lactating mothers, with 1, 1/2, ¼, and 1/8X MIC of nisin A, vancomycin and their combination for 24 h prior to biofilm formation. The amount of biofilm was quantified by measuring the OD595 of crystal violet dissolved in acetic acid. The graphs represent mean values from three biological replicates with the standard deviations. Asterisks indicate statistically significant differences between treatments and between treatments and control for each strain (* = p ≤ 0.05; ** = p ≤ 0.01, and *** = p ≤ 0.001). (TIF) [file pone.0233284.s001.tif]

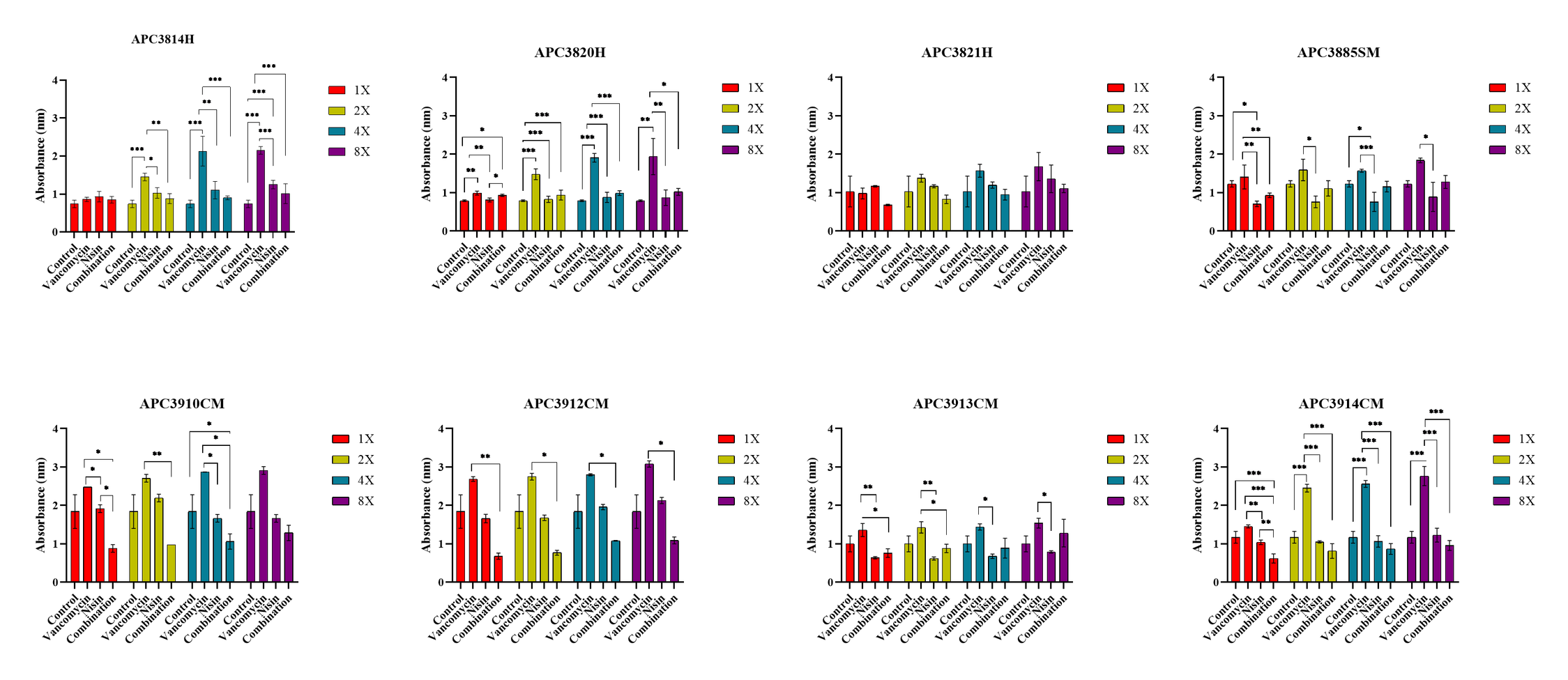

Supplement: S2 Fig — S. aureus strains, isolated from healthy (H), subclinical (SM), and clinical mastitic (SM) lactating mothers, with 1, 2, 4 and 8X MIC of nisin A, vancomycin and their combination for 24h as evaluated by crystal violet straining. The amount of biofilm was evaluated by measuring the OD595 of crystal violet dissolved in acetic acid. The graphs represent mean values from three biological replicates with standard deviations. Asterisks indicate statistically significant differences between treatments and between treatments and control for each strain (* = p ≤ 0.05; ** = p ≤ 0.01, and *** = p ≤ 0.001). (TIF) [file pone.0233284.s002.tif]

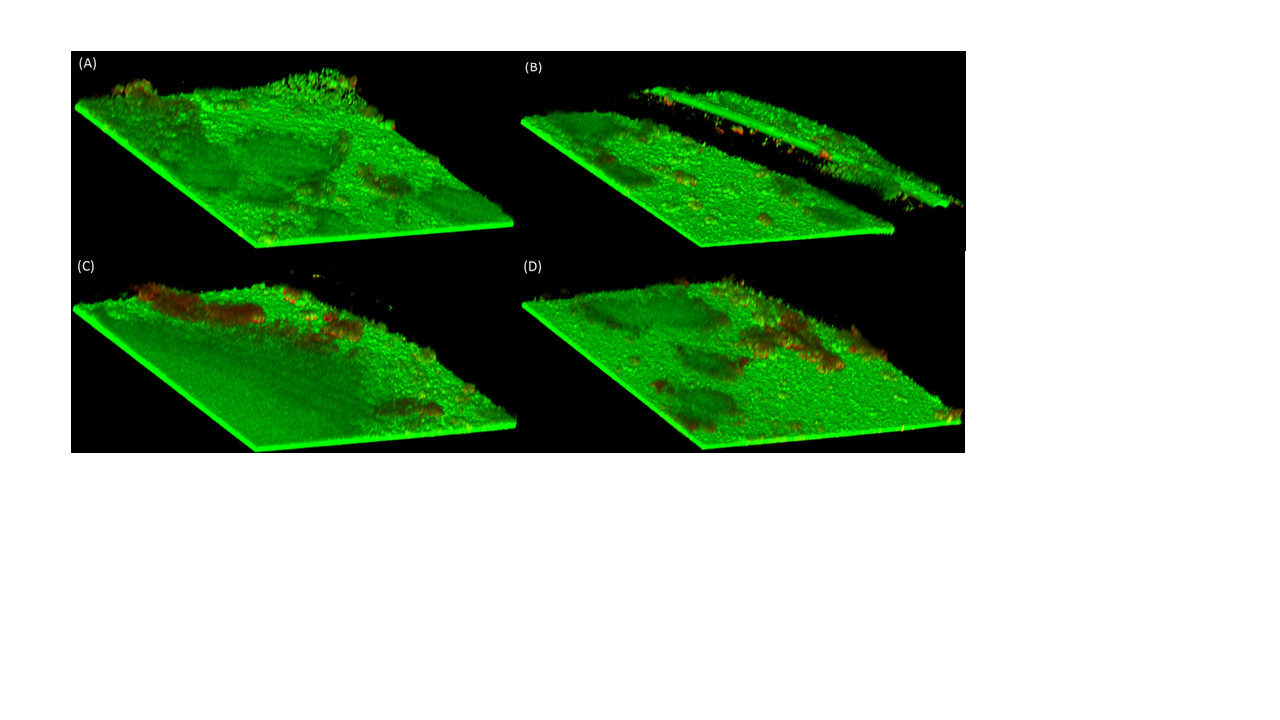

Supplement: S3 Fig — The biofilm was assayed for inhibition after 24 h of treatment; (A) untreated control, (B) 1X nisin A, (C) 1X vancomycin and (D) 1X nisin A + 1X vancomycin. The images were acquired from the edge of the well. (TIF) [file pone.0233284.s003.tif]

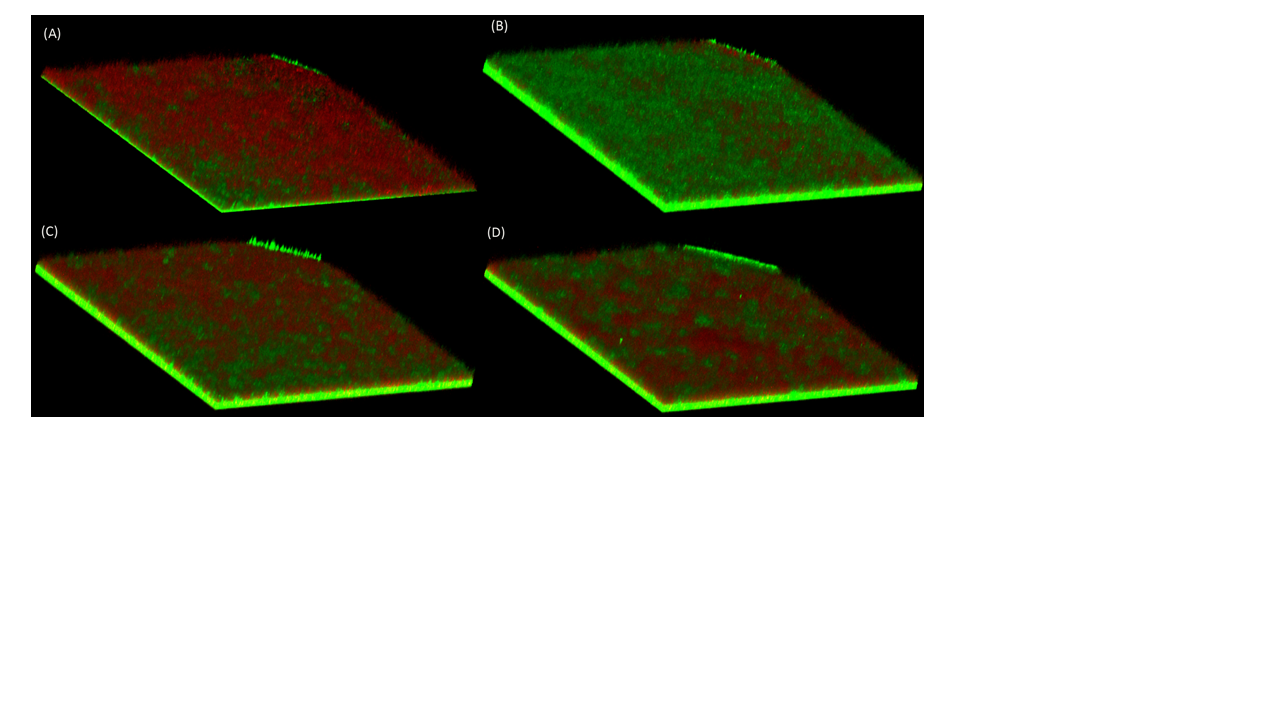

Supplement: S4 Fig — The biofilm was assayed for eradication after 24 h of treatment; (A) untreated control, (B) 1X nisin A, (C) 1X vancomycin and (D) 1X nisin A + 1X vancomycin. The images were acquired from the edge of the well. (TIF) [file pone.0233284.s004.tif]

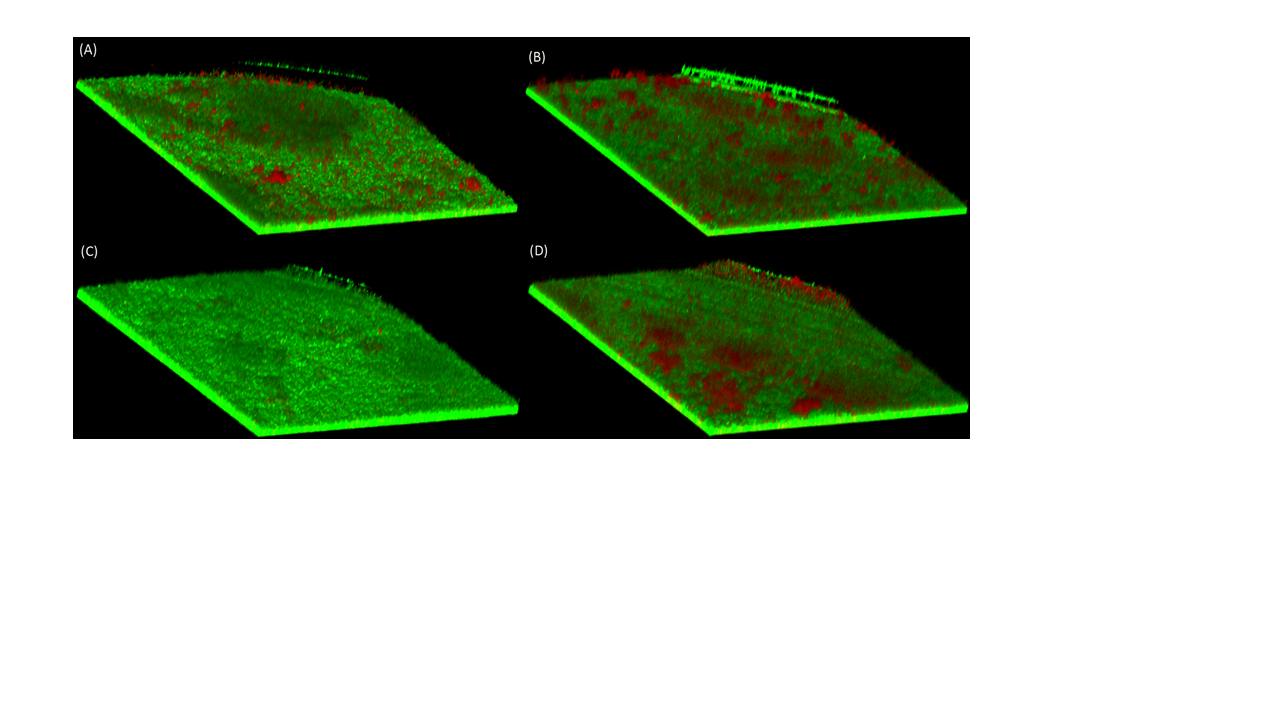

Supplement: S5 Fig — The biofilm was assayed for eradication after 24 h of treatment; (A) untreated control, (B) 1X nisin A, (C) 1X vancomycin and (D) 1X nisin A + 1X vancomycin. The images were acquired from the edge of the well. (TIF) [file pone.0233284.s005.tif]

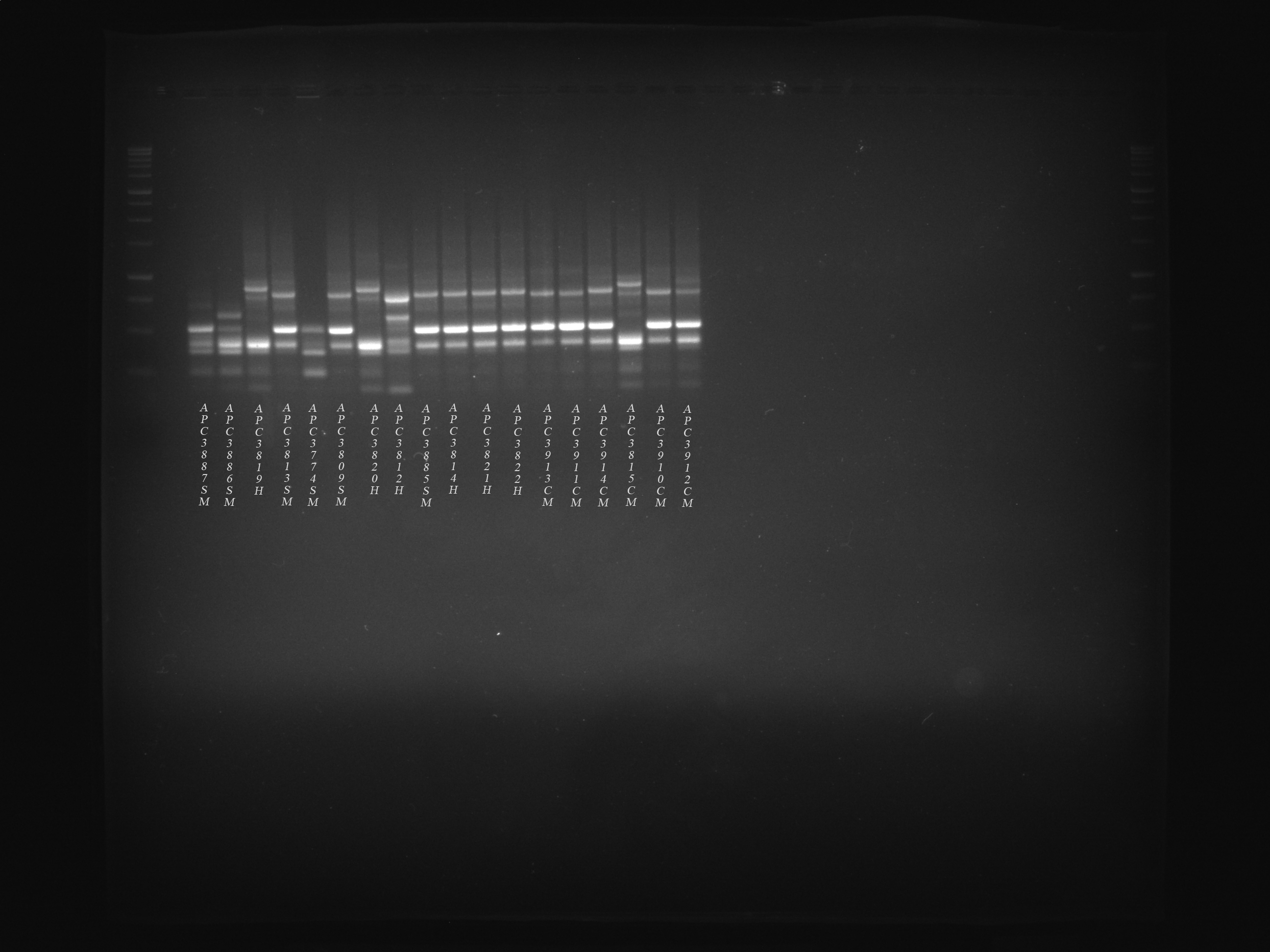

Supplement: S1 Raw images — (TIF) [file pone.0233284.s006.tif]
